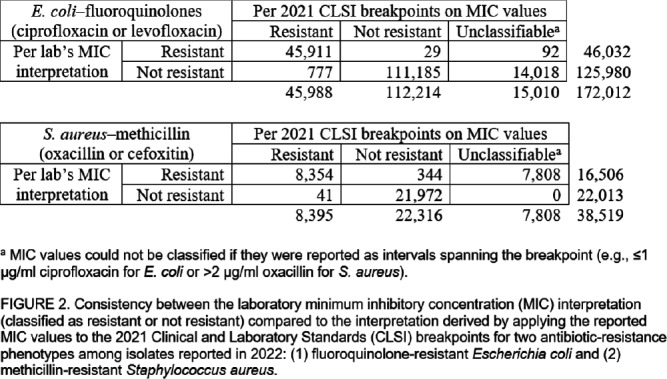# Evaluation of minimum inhibitory concentration data in National Healthcare Safety Network’s Antimicrobial Resistance Option

**DOI:** 10.1017/ash.2024.140

**Published:** 2024-09-16

**Authors:** Mohsin Ali, Amy Webb, Luting Xue, Hsiu Wu

**Affiliations:** Centers for Disease Control and Prevention (CDC); CDC Contractor With Lantana Consulting Group

## Abstract

**Background:** Clinical laboratories perform antimicrobial susceptibility testing (AST) primarily by determining the minimum inhibitory concentration (MIC) for an organism–antimicrobial combination and comparing it with established breakpoints to generate interpretations. The Antimicrobial Resistance (AR) Option of CDC’s National Healthcare Safety Network (NHSN) permits hospitals to submit clinical isolate AST data, including test values and interpretations (Figure 1). The Clinical and Laboratory Standards Institute (CLSI) periodically revises breakpoints, but their adoption by clinical laboratories can be delayed, potentially affecting national AR surveillance data accuracy. Using MIC values, instead of clinical laboratory interpretations, can improve surveillance data accuracy and overcome misclassification due to delayed uptake of revised breakpoints. We evaluated the completeness and consistency of MIC data submitted to the AR Option for fluoroquinolone-resistant Escherichia coli and methicillin-resistant Staphylococcus aureus (MRSA). **Methods:** We included data on (1) E. coli isolates tested for ciprofloxacin or levofloxacin susceptibility and (2) S. aureus isolates tested for oxacillin or cefoxitin susceptibility in 2022 and reported by October 1, 2023. We evaluated completeness among isolates reporting a final AST interpretation as the proportion of isolates reporting both an MIC value and interpretation. We evaluated consistency using percent agreement comparing the laboratory’s MIC interpretation (classified as resistant or not resistant) with the interpretation derived by applying 2021 CLSI M100 breakpoints to the MIC values reported for the same isolate. **Results:** Across 974 hospitals, fluoroquinolone MICs and interpretations were reported for 172,012/393,359 E. coli isolates (43.7%), and oxacillin or cefoxitin MICs and interpretations were reported for 38,519/79,372 S. aureus isolates (48.5%). Of isolates with both MIC values and interpretations, 157,902 (91.8%) E. coli and 7,808(79.7%) S. aureus isolates had MICs that could be classified as resistant or non-resistant (i.e., intermediate or susceptible) per CLSI breakpoints (Figure 2). The remaining MICs were unclassifiable (reported as intervals spanning CLSI breakpoints, e.g., ≤1 μg/ml ciprofloxacin for E. coli). Among isolates with classifiable MICs, the agreement between the clinical laboratory and CLSI-based interpretation was 99.5% for E. coli and 99.7% for S. aureus. **Conclusion:** MIC values and interpretations were available for

**Figure:**
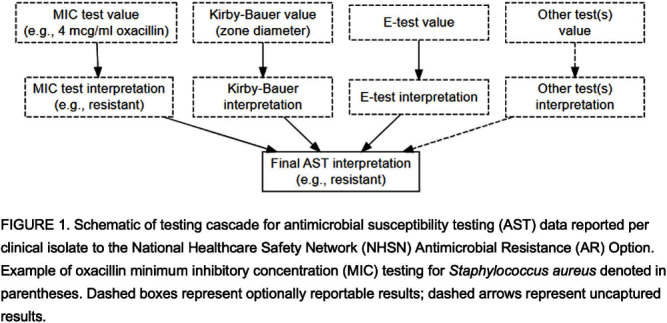

**Figure:**